# Variability of Bile Baseline Excitation-emission Fluorescence of Two Tropical Freshwater Fish Species

**DOI:** 10.1007/s10895-024-03871-x

**Published:** 2024-09-12

**Authors:** Diego Mora-Solarte, Rodrigo Jimenez, Ivonne Calderón-Delgado, Alvaro Duarte-Ruiz, Markus Brinkmann, Yohana Velasco-Santamaría

**Affiliations:** 1 Facultad de Ciencias Agropecuarias y Recursos Naturales, Grupo de Investigación en Biotecnología y Toxicología Acuática y Ambiental (BioTox), Universidad de los Llanos, Villavicencio, Meta 500017 Colombia; 2 Department of Chemical and Environmental Engineering, Universidad Nacional de Colombia – Bogota, Bogota, DC 111321 Colombia; 3 Departamento de Química, Universidad Nacional de Colombia – Bogotá, Bogotá, DC 111321 Colombia; 4 School of Environment and Sustainability and Toxicology Centre and Global Institute for Water Security, University of Saskatchewan, Saskatoon, Saskatchewan, S7N 5B3 Canada

**Keywords:** Water pollution, Metabolites, Fish bile, Fluorescence excitation-emission matrix spectroscopy, Baseline fluorescence, PARAFAC

## Abstract

The quantification of pollutant metabolites in fish bile is an efficient approach to xenobiotic pollution monitoring in freshwaters since these measurements directly address exposure. Fluorescence excitation-emission matrix spectroscopy (EEMS) has demonstrated to be a highly specific and cost-effective technique for polycyclic aromatic hydrocarbon (PAH) and PAH-metabolite identification and quantification. EEMS ability to quantify these compounds strongly depends on the intensity and variability of the bile baseline fluorescence (BBF). We found large differences in BBF among *Aequidens metae* (AME) individuals and of these with *Piaractus orinoquensis* (PIO). Moreover, BBF was large enough that solvent dilutions of over 1:400 were needed to avoid inner filter effects. We used parallel factor analysis (PARAFAC) to model the intra- and inter-species BBF variability. PARAFAC successfully decomposed the EEMS set into three fluorophores present in all samples, although in concentrations spreading over ~ 3 orders of magnitude. One of the factors was identified as tryptophan. Tryptophan and Factor 2 were covariant and much more abundant in AME than in PIO, while Factor 3 was ~ 6 times more abundant in PIO than in AME. Also, tryptophan was ~ 10x more abundant in AME specimens immediately caught in rivers than in their laboratory-adapted peers. The PARAFAC decomposition effectiveness was confirmed by the positive proportionality of scores to dilution ratios. A large inner filter indicates that Factor 2 is as strong a light absorber as tryptophan. Our results stress the need to include bile matrix variable components for the detection and quantification of pollutant metabolites using PARAFAC.

**Supplementary Information:**

The online version contains supplementary material available at 10.1007/s10895-024-03871-x.

## Introduction

Oil reservoirs frequently contain large volumes of associated (fossil) groundwater. Oil production requires the extraction also of associated water, which is either disposed of or reinjected for reservoir pressure maintenance. The Colombian regulations require oil producers to treat associated water even for reinjection. Nevertheless, significant volumes of residual oil end up disposed of in seasonally low-flowrate rivers as associated water volumes are very large and discharge standards and enforcement are not tight enough. More than two-thirds of Colombia’s oil is produced in the Orinoco River watershed (also known as *Orinoquia* or *Llanos* in Spanish). This is a megadiverse region [1], for which the ecotoxicological impacts of oil pollution, particularly on native species [2–9], are of high concern. The average volume ratio of associated water to oil extracted in this region is ~ 17 to 1 [10, 11]. Moreover, a large fraction of oil produced in this region comes from small fields (16–160 m^3^ oil field^− 1^ day^− 1^; 0.1-1 thousand US oil barrels oil field^− 1^ day^− 1^), which use rather simple wastewater treatment technologies [12]. Due to their large number, small fields are more difficult to manage, inspect and control than a small number of large fields. Although water treatment plants are in place and operational, we have frequently observed and identified crude oil films and particles deposited on riverbeds during our fish sampling field campaigns and a strong hydrocarbon smell (Figure S1).

Besides acute exposure of aquatic organisms to oil pollutants, e.g., due to production surges, oil deposits in river sediments can be long-lived, causing chronic exposure. Polycyclic aromatic hydrocarbons (PAHs) are of particular concern among oil pollutants due to their persistence, bioaccumulation, toxicity, and cancerogenic nature [13]. PAH sorption on river sediments is a complex process [14], due to which aquatic organisms exposure can occur even when oil in water concentrations are very small.

Linking sources to effects, i.e., establishing a “bridge of evidence”, is a chief goal of environmental research, yet usually a very challenging one. Source attribution can become blurred by ecosystem biotransformation and biological adaptation processes [15, 16]. The presence of pollutants or their metabolites in tissues of aquatic organisms constitutes major, indisputable evidence for pollutant exposure, sometimes traceable to sources. Bioindicator organisms evidence pollution impacts through their physiological responses [17]. In recent years, fish bile has been put forward as a contamination biomarker compartment, as the gallbladder is a reservoir for xenobiotics and intermediate compounds that result from organism biotransformation and detoxification processes. Therefore, the detection of pollutants or their metabolites in bile is a direct measure of exposure [18, 19].

The monitoring of hydrocarbon pollution in various biotic and abiotic matrices can be performed regularly with high specificity and precision at the sub-part-per-billion (ppb) levels with analytical techniques that are rather complex, expensive, and slow [19], e.g., gas chromatography coupled with mass spectroscopy. Various fluorescence techniques have been put forward as complementary to or potentially even as replacements of these established analytical techniques [20–23]. Although fluorometers can be highly sensitive, comparatively inexpensive, reasonably selective, and even portable, thus excellent for screening and detection purposes, direct fluorometric measurements are not intrinsically specific. Fluorescence measurements of complex mixtures require calibration and chemometrics to be employed as quantitative analytical techniques. Specificity can be improved dramatically by measuring fluorescence at multiple excitation and emission wavelengths. The resulting fluorescence spectral surface or excitation-emission matrix (EEM) constitutes a fluorometric fingerprint of the sample. The challenge is then to deconvolute a spectral surface into its contributing fluorophores and quantify their concentrations [23–26].

Excitation-emission matrix spectroscopy (EEMS) has proven to be a highly cost-effective and quite selective technique for PAH and PAH-metabolite detection, screening, and quantification when used in conjunction with linear and nonlinear chemometric methods [26–29]. An observed spectral surface is the combination product of fluorescence from all the effectively excited fluorophores in the mixture along with first- and second-order scattering, among other non-linear effects, including fluorometer spectral convolution. Moreover, fluorescence is linearly proportional to concentration and combines linearly only in the thin limit, which usually equates to very low concentrations for strong fluorophores. Outside this region, the so-called inner filter effect, a concentration-fluorescence non-linearity, can significantly bias fluorophore quantification. Several methods have been applied to mend these problems, with varying levels of success [29–32]. The fluorescence baseline, i.e., the fluorescence of non-targeted fluorophores, such as solvents, and especially of complex fluorophore mixtures in natural matrices, such as fish bile, is a remaining, usually non-trivial issue, which can significantly hinder the quantification of targeted fluorophores. Also, solvent-water interactions can cause second-order fluorescence effects. The fluorescence baseline is even more complex for natural matrices due to fluorophore content intra- and inter-species variability. Thus, for instance in the case of exposure to xenobiotic compounds, samples from unexposed individuals might not be a suitable baseline due to differences among individuals of the same species. Therefore, a simple spectral subtraction might not suffice to remove a fluorescence baseline, particularly of natural matrices.

Here we report significant intra- and inter-species variability of bile fluorescence among laboratory-raised freshwater fish (*Aequidens metae* and *Piaractus orinoquensis*) and individuals of *A. metae* captured in rivers compared with their lab peers. These are freshwater fish species native of the Orinoquia region. We also assessed the ability of the Parallel Factor Analysis (PARAFAC) model to capture this variability, including for fish in rivers with and without apparent contamination.

## Materials and Methods

Our investigation included fluorometer characterization and optimization, and sampling and analysis of the bile fluorescence of two species of native fish. Fish were caught alive in (1) pollution-free ponds; (2) an apparently effluent-free river; and (3) a river impacted by oil production wastewater treatment outflow. Fish from pollution-free ponds were transferred to and maintained alive in laboratory conditions for baseline and exposure analysis purposes. The results of fish exposure to PAH under lab conditions has been reported elsewhere [33].

### Pollution-Free Pond Fish Samples

Live *A. metae* (AME – Yellow Acara or *Luminosa* in Spanish) specimens were procured from a local fisherman, who catches them in pollution-free ponds in the Province of Meta, Colombia. These ponds are small reservoirs that form in low-flow rivers (or *caños* in Spanish) during the rainy season. Right after, AME were acclimatized and quarantined for 30 days in 250-L plastic tanks. This procedure was conducted to adapt the fish to confinement and to flush them from any xenobiotics potentially present in their original habitat. After this period, fish were maintained in a 20-L constant aeration aquarium for 10 adaptation days prior sampling. All our procedures were bound to the Universidad de los Llanos’ Bioethics Committee regulations (https://bit.ly/41bWQM9). The following physicochemical water quality parameters were maintained during the laboratory adaptation period: temperature 24 ± 2 ºC; photoperiod 12 h light, 12 h dark; pH 6.9 ± 0.1; and dissolved oxygen 6.7 ± 0.2 mg L^− 1^.

AME individuals weighed 9.8 ± 0.3 g and had a total length of 5.8 ± 0.1 cm. Their gallbladder diameter was approximately 1–4 mm, thus with volumes ranging from 5 to 12 µL. Considering that the fluorescence cuvette required a diluted 4-mL sample, the bile volume collected seemed insufficient for individual analysis, so, bile samples were pooled from the gallbladders of 5 individuals. Composite bile sample volumes of approximately 25–35 µL were finally obtained. Our measurements later revealed that AME bile was fluorescent enough that pooling might have been unnecessary in some cases.

Fish were euthanized by severance of their spinal cord to obtain bile samples. Gallbladders were immediately removed, and bile samples obtained using a micropipette. Three pooled bile samples, each one from five fish individuals, were obtained and collected in 500-µL reaction tubes. Upon extraction, each tube was homogenized in a vortex shaker for 30 s and stored at -20ºC until their subsequent analysis [34].

### Reference and Polluted Rivers Samples

For comparison and potential contamination identification purposes, *A. metae* (AME) specimens were also captured in a river apparently free from pollution sources (*Caño Cuncia* – 4° 3’ 7” N, 73° 43’ 46” W), hereafter referred to as “REFERENCE” river (REF), and a river in which oil production wastewater treatment outflow is discharged (*Río Acacias* – 3° 57’ 12.84’’N, 73° 40’ 6’’ W), hereafter referred to as “POLLUTED” river (POLLUT).

Eight and six individuals were captured in the POLLUT and REF rivers, respectively. Individuals weighed 12.1 ± 0.8 g and were 6.3 ± 0.1 cm long (total length) on average. POLLUT river fish had lower bile volume that REF river fish. Nonetheless, in both cases, individual bile volume was insufficient for analysis. Only one combined sample was obtained from each river. The total bile volume was ~ 16 µL per site. Samples were obtained in the same way than for laboratory fish. No significant differences were found between the POLLUT and REF rivers water basic physical-chemical parameters, temperature (27.6 ± 0.6 ºC), pH (7.1 ± 0.1) and dissolved oxygen (7.4 ± 0.4 mg L^− 1^).

### Inter-Species Comparison

We also compared the AME samples to bile samples of Cachama Blanca specimens (*P. orinoquensis*, PIO) obtained from a previous study [5]. As this was an exposure investigation, we measured the fluorescence of two bile samples from the PIO control group. These samples were stored at -20 °C for two years. According to Aas, et al. [34], metabolites in bile can be preserved for years without alteration if samples area stored at low temperature (-20 ° C to -80 ° C).

### Solvent Selection and Fluorometer Optimization

We used a Cary Eclipse double monochromator fluorometer (Model G9800A – Fluorescence, Agilent Technologies), operating with its own software (version 1.2 (147)). To optimize its performance, we evaluated and selected values for the following measurement variables and parameters:

1. Averaging time and nominal signal-to-noise ratio (SNR): these parameters control the fluorescence intensity and noise at given excitation and emission wavelengths but also the time required to measure the resulting spectral surface (EEM), built from hundreds of individual measurements. We assessed three possible individual measurement averaging times (0.1 s, 0.5 s, and 1 s). We also evaluated the actual measurement quality effects of three distinct nominal SNRs (5, 100, and 1000). The actual SNR is nominal as it is internally calculated and controlled by the instrument with no user intervention besides the setting itself.
2. Slit aperture: this parameter controls both the fluorescence signal intensity and its spectral resolution. Three different slit-aperture equivalent spectral resolutions (1.5 nm, 2.5 nm, and 5 nm) were assessed to determine an appropriate trade-off value.
3. Nominal spectral resolution: spectral lines and bands in condensed phase (liquid or solid) absorption and fluorescence spectra are usually tightly packed, i.e., highly convolved thus intrinsically broad, so they usually cannot be resolved, which entails that high spectral resolution is usually unnecessary. Therefore, the time spent measuring fluorescence over a dense excitation-emission sampling grid might be better used for a longer individual measurement averaging time. For parameter optimization purposes, we assessed three different excitation and emission wavelength step values, 0.2 nm, 2 nm, and 5 nm.
4. Type and purity of solvent: we tested methanol, isopropanol, ethanol, and 1:1 ethanol-water as potential solvents. The solvent selection criteria were the absence of interfering fluorescence in spectral regions of relevance for fish bile and potential metabolites, and evaporation rate using vapor pressure at room temperature as proxy. The use of the 1:1 (v/v) ethanol-water solution was previously reported [35]. We also assessed the analytical impact of solvent purity.

### Sample Preparation and Measurement

As discussed below, the spectral data analysis and other considerations lead us to select 1:1 (v/v) ethanol – deionized water as solvent. Bile samples were vortex-mixed for 30 s, then serially diluted in the ethanol-water solution to assess inner filter effects [34, 36]. As bile natural fluorophores were not chemically identified nor quantified in this investigation, we used the dilution factor (*DF*) as a proxy for fluorophore concentration,

1 $$\:DF=\raisebox{1ex}{$c$}\!\left/\:\!\raisebox{-1ex}{${c}_{0}$}\right.=\raisebox{1ex}{${V}_{0}$}\!\left/\:\!\raisebox{-1ex}{$({V}_{0}+{V}_{solvent})$}\right.$$

where *V*_*0*_ is the bile sample volume prior dilution (initial volume), $\:{V}_{solvent}$ is the volume of ethanol-water added for dilution, and $\:{c}_{0}$ and $\:c$ are the concentrations of a fluorophore in bile prior dilution and upon dilution, respectively. Although nearly identical at high dilutions, the dilution factor (*DF*) must not be confused with the dilution ratio ($\:{V}_{0}/{V}_{solvent}$). Bile samples were serially diluted and measured from ratios of 1:100 (*DF* = 0.01) or 1:200 (*DF* = 0.005), depending on the bile initial volume, down to ratios at which the fluorescence signal intensity was very low, tipically at *DF* = 0.0003.

Peak excitation and emission spectra, and excitation-emission matrix spectra (EEMS) were derived from scanning mode measurements at excitation and emission wavelength intervals of 190–400 nm and 250–600 nm, respectively. The identification of the peak excitation and emission wavelengths, i.e., the wavelength pair at which fluorescence is maximum, allows for the calculation of the peak excitation and emission spectra, which are the EEM slices at constant peak emission and constant peak excitation wavelengths, respectively. A total of 7 bile samples (3 laboratory-conditioned AME pooled samples, 2 samples from laboratory-conditioned PIO individuals, and AME pooled samples from individuals captured in clean and polluted rivers, one sample each) were measured, each at six dilution levels on average. Upon optimization, the total number of combinations included 43 bile dilutions, 43 excitation wavelengths, and 81 emission wavelengths, for a total of 146,286 fluorescence measurements.

### Data Processing, Parallel Factor, and Statistical Analysis

Excitation light, and to a lesser extent, emission light, can be significantly scattered in fluorescence measurements. Elastic (Rayleigh, Mie) and inelastic (Raman) scattering effects can severely affect fluorescence spectra. Moreover, grating monochromator excitation light contains small but significant levels of second order, wavelength-doubled diffraction light. Data reduction techniques, such as Parallel Factor Analysis (PARAFAC), are unable to tell fluorescence apart from scattering, thus their results could be highly biased if scattering effects are not removed before data analysis [29, 37]. In the case of PARAFAC, scattering would artificially increase the number of components needed to explain a spectral surface [29]. To mend this, we used the *drEEM* tool (version 0.6.0) and the *smootheem* function [29, 38] to remove four scattering bands prior analysis as follows: first-order Rayleigh (+ 20 nm and − 10 nm, 30 nm total around the Rayleigh signal); second-order Rayleigh (+ 20 nm and − 15 nm, 35 nm total); first-order Raman (+ 5 nm and − 15 nm, 20 nm total); and second-order Raman (+ 10 nm and − 15 nm, 25 nm total).

We chose PARAFAC because of its proven ability to identify common fluorophores (“factors”) among complex mixtures of fluorescent compounds, even when their spectral surfaces appear uncorrelated at first sight. As for other attribution methods, the remaining challenge is to trace factors back to specific fluorescent constituents [24, 29]. PARAFAC represents a correlated, or likely correlated, EEM set as a 3-D tensor product with indices I, J, and K (Eq. 2) as follows:

2 $$\eqalign{ {X_{ijk}} = & \mathop \sum \limits_{f = 1}^F {a_{if}}{b_{jf}}{c_{kf}} + {e_{ijk}}\,{\text{for}}\,i = 1 \ldots I, \cr & j = 1 \ldots J,k = 1 \ldots K \cr}$$

where $\:{X}_{ijk}$ is the fluorescence intensity for the *i*-th sample at the *j*-th emission wavelength and the *k*-th excitation wavelength and $\:{e}_{ijk}$ are the fitting residuals, which should ideally contain only uncorrelated, featureless noise. Each factor *f* corresponds to a PARAFAC component, to which an *a*-value (score) is computed for each sample. Scores to a factor are expectedly proportional to the fluorophore (factor) concentrations in the samples. To model their spectral shape, each factor (component) also has *J b*-values, corresponding to the estimated loadings for each emission wavelength, as well as *K c*-values, corresponding to the estimated loadings for each excitation wavelength.

PARAFAC assumes linearity between fluorophore concentration, of which $\:{a}_{i,f}$ is a proxy, and fluorescence signal intensity (modeled as the *b* times *c* product), i.e., it cannot account for inner filter effects. Therefore, we used only diluted enough samples ($\:DF\le\:0.0025$) for this analysis with some exceptions.

Before applying PARAFAC, we subtracted the 1:1 ethanol-water blank EEM spectral surface from all the samples. It might appear simpler just to include the ethanol-water blank as a sample and let the PARAFAC fitting software identify it as a factor [38]. Our tests showed that this procedure is ineffective and causes an artifact as it artificially adds degrees of freedom to the fitting problem. The PARAFAC model was fitted using the N-way tool [38] (version 3.30) in MATLAB (version 2018b). Non-negative restrictions were added to avoid model overfit to small negative values resulting from blank subtraction and the scattering removal procedure. Parameters such as core consistency and residual sum of squares (RSS) were used to assess the data reduction performance and the appropriate number of factors. These zero-dimensional indicators are frequently insufficient to spot spectrally localized issues and to assess the ability of PARAFAC to identify new factors. To deepen our analysis, we coded and used a modeled-to-observed spectral surface discrepancy routine, which allowed us to calculate and visualize local bias, and local root-mean-square error (RMSE) and standard deviation (SD) on the excitation and emission wavelength space. These last two bivariate discrepancy indicators were calculated using a ± 10% 2-D moving window. Considering that both, observed and modeled fluorescence spectral surfaces bear uncertainly, reduced major axis (RMA) regression was applied to fit modeled to observed surfaces, instead of simple linear regression. Fitted offset, slope, and Pearson coefficient (*r*^*2*^) were thus RMA’s.

## Results and Discussion

### Detector Optimization

Fluorometer trade-off parameters were derived from 3-level measurements of isopropanol and *Piaractus orinoquensis* bile (*DF* = 1.12 × 10^− 3^). Various combinations of slit width, nominal SNR, averaging time and spectral resolution were tested. The main conclusion of this optimization was that SNR was dominant over averaging time, since the setting of this parameter forced the equipment to reach a chosen SNR regardless of the predefined averaging time for both samples. However, the comparison between nominal averaging times show that time longer than 10 s degraded the matrix resolution. Therefore, a SNR of 1000 and an averaging time of 1 s were set for the rest of the measurements.

Regarding slit width and nominal spectral resolution, based on other bile fluorescence studies [26], we anticipated that the finest structure to be resolved was ~ 5 nm wide (FWHM). Due to time and fluorometer usage constraints, we set our resolution at 5 nm. Our isopropanol measurements (Figure S2) revealed that a slit width narrower than 5 nm provided no advantage in terms of actual spectral resolution at a nominal spectral resolution of 5 nm. We thus set the slit width at 5 nm to optimize the fluorescence signal.

### Solvent Type and Purity Effects

Isopropanol was discarded as solvent due to its high fluorescence (Figure S3). Various ethanol and methanol brands and grades were evaluated as bile solvents.

Several authors have reported that chromatography-grade methanol properly solubilize bile and reduce inner filter and quenching, among other effects [26, 39, 40]. The fluorescence of various methanol brands and grades was thus compared considering potential impurities and their spectral effects. Methanol, ethanol and ethanol-water EEMS were obtained in scanning mode over the 190–400 nm excitation interval while observing emission along the 250–600 nm interval. Table 1 summarizes the excitation and emission intervals where significant fluorescence was observed, the fluorescence peak wavelength pairs (excitation, emission) and their intensity for four different commercial methanol options. As expected, the fluorescence interval of analytical-grade methanol was wider than for chromatography-grade (HPLC) methanol (Figure S4). Overall, the commercial methanol fluorescence fingerprint was wider and higher than expected with a large inter-brand variability, likely due to hydrocarbon contamination. Methanol fluorescence was high compared with the bile fluorescence baseline (BFB) of both fish species (Fig. 2), and potential overlapping. Moreover, although one of the HPLC-grade methanol brands yielded a significantly lower florescence, its price and the volumes required to reach inner filter free concentrations made it economically unfeasible for us. For this reason, we assessed ethanol (EtOH) as a solvent, which had been previously reported as a good bile and hydrocarbon diluent, with which inner filter effects may be reduced [20, 35, 41]. An important, additional consideration is that the volatility (vapor pressure) of EtOH at room temperature is a factor two smaller than for methanol, which implies that potential biases due to solvent loss during long bile dilution-measurement cycles were significantly lower for ethanol than would have been for methanol.

**Table 1:** Fluorescence characterization of various tested methanol and ethanol grades and brands

	Grade	Brand	λex Excitation wavelength interval [nm]	λem Emission wavelength interval [nm]	Peak excitation wavelength [nm]	Peak emission wavelength [nm]	Max fluorescence intensity [AU]
Methanol	HPLC (CAS N° 67-58-1, Ref. 9093-03)	J.T. Baker	200–225	270–315 / 560–590	215 215	285 565	59.1 26.9
	Liquid chromatography LiChrosolv^®^ (CAS N° 67-56-1, Ref. 1.06018)	Merck	205–220	270–305	215	295	13.9
	For analysis, ACS (CAS N° 67-56-1, Ref. 131,091)	Panreac	195–290	270–360	215	290	66.8
	For analysis EMSURE^®^ ACS (CAS N° 67-56-1, Ref. 1.06009)	Merck	220–300	320–405	250	365	691.2
Ethanol	Absolute for analysis ACS (CAS N° 64-17-5,Ref N°1.00983)	Merck	200–220	270–310	210	290	21.1
	Absolute 99.5%(NO CAS N°, N°, Ref. AE99)	Chemi	290–330	310–405	300	330	20.8
	For Molecular Biology, 190 proof (CAS N° 64-17-5, Ref E7148)	Sigma Aldrich	200–235	280–335	225	305	27.6

Most of the evaluated EtOH brands and types showed lower fluorescence than for methanol (Table 1). However, the EtOH brand and grade with lowest fluorescence intensity (Chemi, analytical grade) showed strong light absorption in the 190–290 nm interval. Its low fluorescence can be thus explained by strong inner filter effects. This suggest that this brand and grade contained enough residual hydrocarbons to make it unsuitable as bile fluorescence solvent (Figure S5). The mean fluorescence of the two remaining EtOH options (Merck and Sigma brands) was statistically quite similar (t-student, *p* > 0.05). We finally chose the Merck ethanol as solvent due to its narrower excitation and emission intervals.

**Fig. 1:**
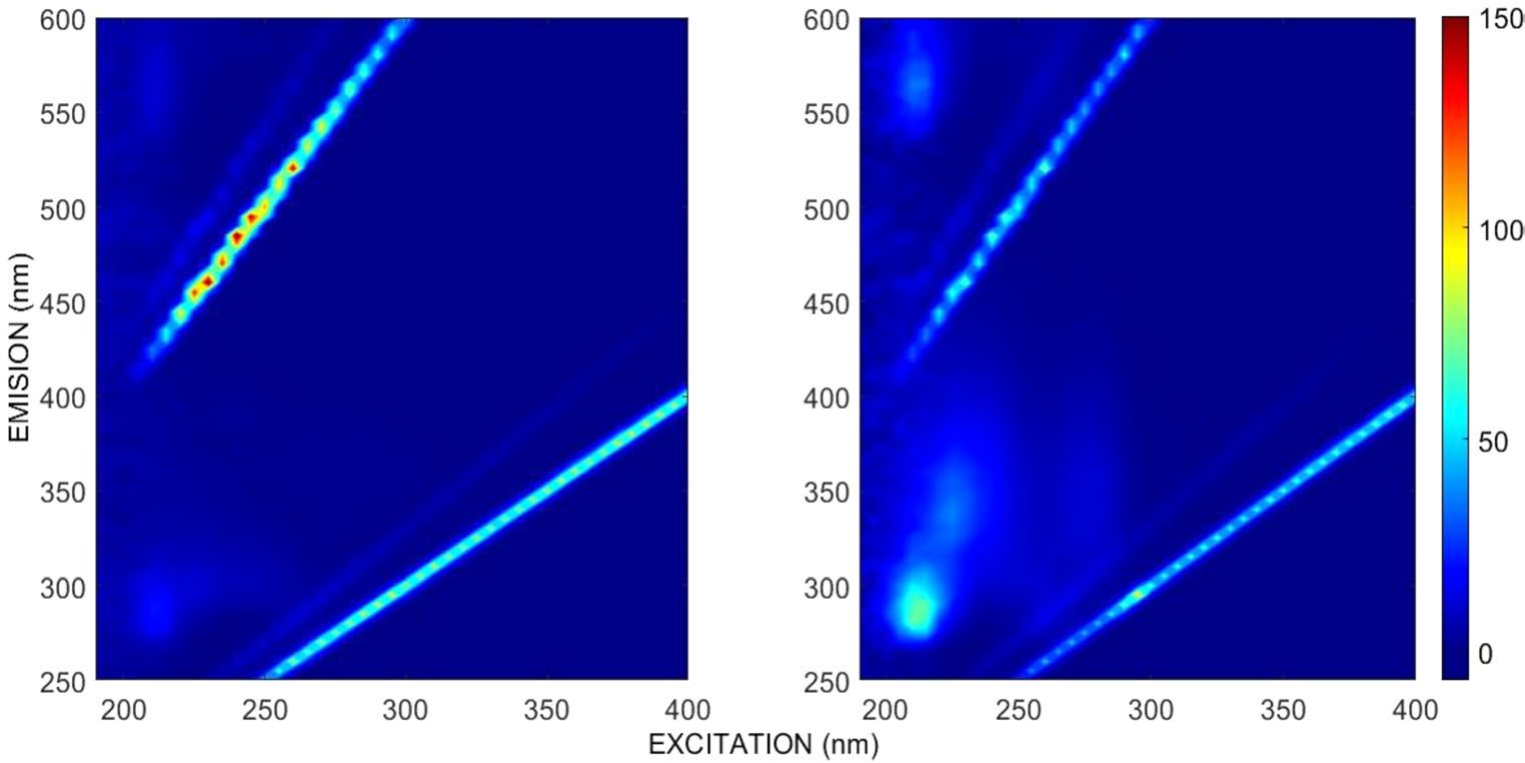
Selected ethanol EEMS. Left: Pure ethanol (EX 210 nm, EM 290 nm). Right: 1:1 (v/v) solution of ethanol in deionized water (EX 210 nm, EM 280 nm)

We quickly realized that the volatility of bile samples diluted in pure EtOH was too high considering the long fluorescence EEMS measurement times and the number of dilution-measurement cycles. It would have been even higher with methanol as solvent [42]. To appropriately reduce sample volatility and ensure bile concentration stability, we adopted the 1:1 (v/v) ethanol to deionized water mixture (EtOH-H_2_O) as bile solvent for the rest of the measurements. The EEMS of pure EtOH and the 1:1 EtOH-H_2_O mixture are shown in Fig. 1. The first and second order Rayleigh and Raman scattering are also clearly observed (glowing diagonal lines).

Pure EtOH displayed a rather shallow fluorescence peak of less than 30 AU at excitation and emission wavelengths of 210 nm and 290 nm, respectively. In contrast, the 1:1 (v/v) ethanol – deionized water solution showed a much larger fluorescence intensity (71 AU) at the same excitation wavelength (210 nm) but at a slightly blue-shifted emission wavelength (280 nm).

Jia, et al. [43] investigated the EtOH-H_2_O system fluorescence in the 280–450 nm interval, excited with 240 nm light. They found a monotonic, sigmoidal increase of fluorescence at EtOH fractions of up to ~ 70%, followed by an inner filter like behavior at larger fractions. They also found that the fluorescence spectra at a given EtOH volume fraction changed over time during an “incubation” period of < 7 days. This was attributed to heterogeneity at the molecular level and the formation period of three spectrally identified hydrogen-bounded clusters, of which the most stable, (H_2_O)m(EtOH)n (50–75% v/v), was predominant at our volume fraction (50%). As per their results, our EtOH: H_2_O solvent fluorescence should not change more than ±8% during the incubation period and negligibly thereafter. Upon solvent selection, our chief goal was to reduce the confounding factors for PARAFAC. Therefore, the fluorescence of the 1:1 v/v EtOH-H_2_O matrix, i.e., our blank, was simply subtracted from that of the bile samples [44].

### Scattering Signal Removal

First and second order Rayleigh and Raman scattering were evident on the EEM spectra of the solvent (Fig. 1) and of diluted bile samples [29] (Figure S6). Moreover, the mathematical subtraction of the chosen solvent (1:1 v/v EtOH-H_2_O) EEM spectrum was insufficient to fully remove the scattering signal from the bile EEM spectra, particularly at high bile concentrations. This suggested that some of the scattering observed in bile samples was not molecular but due to suspended bile microparticles (Mie scattering). Although bile samples were translucent, some turbidity was observed naked eye in the concentrated bile samples, while no turbidity was observed in the solvent. This was corroborated with a dynamic/electrophoretic light scattering particle size measurement (Zetasizer Nano-ZS, Malvern), which showed a suspended particle size dominant mode of 100–300 nm in a bile sample at *DF* = 3.1⋅10^− 4^ (FigureS7).

The residual scattering upon blank subtraction was high enough to hinder the analysis of fluorescence surfaces and to overestimate, bias and even create spurious factors in the PARAFAC decomposition. To prevent this, we removed the residual scattering signals from the EEM spectra using the drEEM tool (version 0.6.0), including the *smootheem* function to interpolate over the spectral surface gaps resulting from scattering removal (Figure S6) [29]. The irreducible Raman and Rayleigh scattering was lower than 2 AU after this processing. Moreover, PARAFAC converged faster after scattering removal and the subsequent analysis had clearer significance [32].

### Inner Filter Effects

Fluorescence is expected to monotonically increase with fluorophore concentration, linearly within the thin limit and asymptotically thereafter, up to an absorbance level from which it monotonically decreases with concentration due to a complex interplay of spectroscopic, optical, and chemical effects known as inner filter [45, 46]. The dilution curves (fluorescence as a function of dilution factor, *DF*) of *Aequidens metae* clearly showed this saturation- then parabolic-like behavior. For fluorescence factor decomposition purposes, PARAFAC assumes that a positive linear relationship exists between fluorophore concentration and fluorescence, but this is valid only in the “thin limit”, i.e., at low absorbances. Instead of attempting to correct the fluorescence surfaces for inner filter effects [29–31], i.e., for deviations from the linear dependence of fluorescence on concentration, we preferred to simply exclude from the PARAFAC analysis the spectral surfaces that were clearly affected by inner filter effects, mainly because PARAFAC would have been unable to interpret this non-linearity, thus leading to poor and heterogenous stability results [47].

*A. metae* bile sample dilution curves consistently showed maximum fluorescence intensities at dilutions within 1:400 and 1:800 (*DF =* 1.25⋅10^− 3^ – 2.5⋅10^− 3^). Inner filter effects were absolutely evident at higher bile concentrations (Figure S8) [48]. All *A. metae* bile samples showed inner filter effects in their main and secondary fluorescence peaks, but no inner filter effects were observed in any *P. orinoquensis* individual bile samples at comparable fluorescence intensities, i.e., fluorescence behaved linearly upon dilution for *P. orinoquensis* individual bile samples. This suggests that, *P. orinoquensis* fluorophores are in lower concentrations or that they are less efficient light absorbers than *A. metae* but eventually have higher fluorescence quantum yields.

### Inter- and Intra- Species Variability

**Fig. 2:**
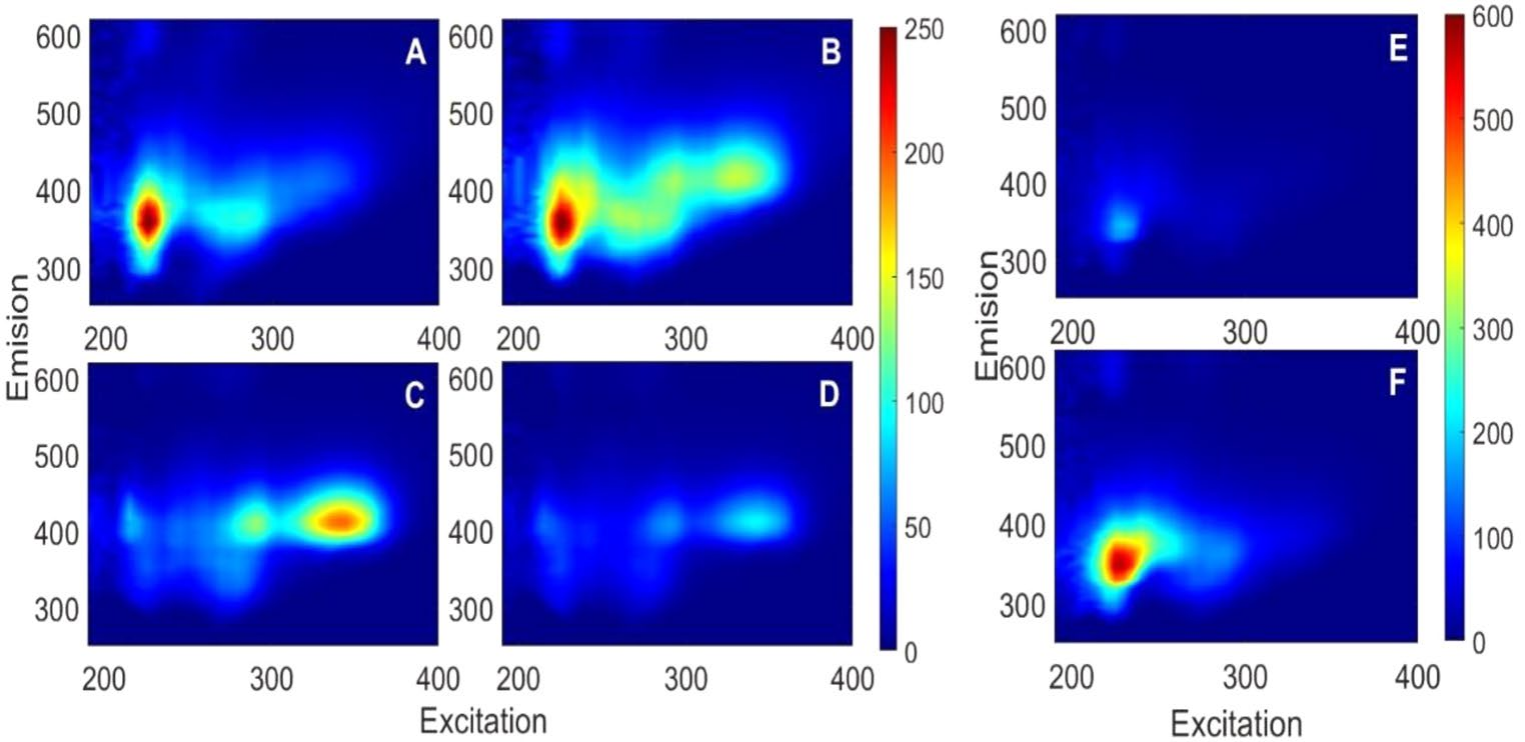
EEMS of laboratory-adapted *A. metae* (panels **A** and **B**) and laboratory-adapted *P. orinoquensis* (panels **C** and **D**) bile samples at 1:800 dilution (*DF* = 1.25 ⋅10^− 3^), and bile samples of *A. metae* caught in apparently effluent-free and polluted rivers (panels **E** and **F**, respectively) at 1:6400 dilution (*DF* = 1.56 ⋅10^− 4^). Note that the BBF of the polluted river specimens is larger than that of their lab peers even when 8 times more diluted

The bile EEMS of the two Colombian native fish species appeared to be very different (Fig. 2). The fluorescence peak intensity was a factor 1.86 times higher for *A. metae* (AME) compared to *P. orinoquensis* (PIO) at the same dilution factor (1:800), which is consistent with undetectable inner filter effects for PIO. Moreover, the fluorescence peaks appeared at completely different excitation and emission wavelengths, which suggest distinct main fluorophore families in each species. AME highest fluorescence intensity was observed at λ_Ex_ = 225 nm and λ_Em_ = 360 nm for three pooled samples from laboratory (Fig. 2-A, 2-B, and 2-C), while for PIO the peak was observed at λ_Ex_ = 345 nm and λ_Em_ = 410 nm (Fig.2-D and 2-E). Finally, significant differences in intensity and spectral surface were observed among individuals of the same species, despite being maintained under standard water quality and food-controlled conditions. This points to the physiological state of each individual organism as a controlling factor of its bile fluorophore concentration. It is known that stress conditions resulting from confinement, dominance, or voracity can disrupt the physiological function of bile acids or salt production [49, 50]. These differences were observed among individuals and species at the same dilution ratio.

**Fig. 3:**
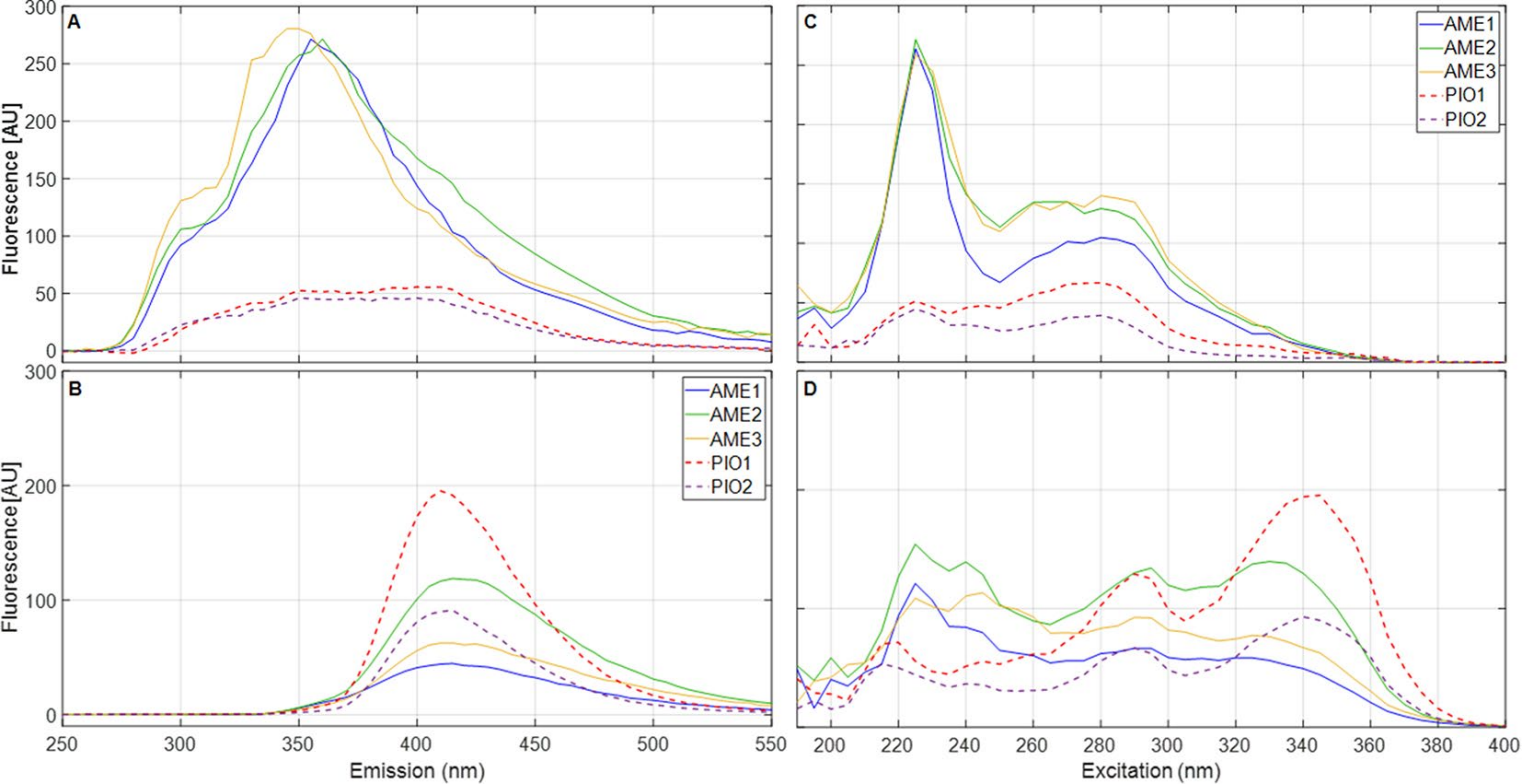
Excitation and emission spectra of *A. metae* (AME) and *P. Orinoquensis* (PIO) bile samples at a 1:800 dilution ratio (*DF* = 1.25⋅10^− 3^). Emission spectra are shown at their peak excitation wavelengths of 225 nm (panel **A**) and 345 nm (panel **C**). Excitation spectra are shown at their peak emission wavelengths of 360 nm (Panel **B**) and 410 nm (Panel **D**)

A deeper spectral analysis showed that the modal spectral peaks in one fish species are actually present in the other, although with a much lower intensity (Fig. 3). This suggest that the apparently distinct spectral surfaces actually resulted from significantly different proportions of fluorophore main groups between the two species and much smaller concentration variations among individuals of the same species [51]. The two main excitation (225 nm and 345 nm – Fig. 3A) and emission peaks (360 nm and 410 nm – Fig. 3B) appeared in all the samples of the two species with comparable peak widths (measured as full width at half maxima – FWHM). These results encouraged the use of PARAFAC for factor identification and data reduction.

### PARAFAC Modeling and Fluorophore Identification

The spectral surfaces were pre-processed to eliminate Rayleigh and Raman scattering as explained above. We initially applied PARAFAC for data reduction to all the *A. metae* samples spectral surfaces without considering inner filter effects. This was made for the subset of serial dilutions of the same sample, and for all the AME samples and their dilutions. As expected, PARAFAC was not suited to model inner filter non-linearities. The reported variabilities were very large, and the fittings were rejected as per their validation parameters. Therefore, relatively concentrated bile samples with evident inner filter effects were discarded for the PARAFAC fitting. Only AME samples diluted either to 1:400, 1:800 or more were included in the PARAFAC analysis.

The total array of the pre-processed data comprised 43 excitation wavelengths, 81 emission wavelengths, and 32 dilutions of fish bile samples, coming from 3 laboratory-conditioned AME pooled samples, 2 samples from laboratory-conditioned PIO individuals, and AME pooled samples from individuals captured in clean and polluted rivers, one sample each. We tested PARAFAC data decompositions into 1 to 7 factors (components) to determine a quasi-optimal number of factors for each case (Table 2). Results with up to four (4) factors are discussed here as additional factors did not improve core consistency. In all cases, a convergence criterion of 10^− 6^ was used and a maximum of 2500 iterations were allowed [44].

No restrictions were initially applied to the fitting model, which led to physically meaningless, unsuitable for interpretation, negative values, either in the scores (proxies of concentrations) or in the loading matrices (*B* and *C*), which define the spectral shape of the factors [44]. In order to improve the stability and significance of the model, non-negativity restrictions were included for the three loading matrices (also called modes) [24]. The resulting core consistency was typically higher than 70%, which indicates a good adjustment of the model (Table 2).

**Table 2:** PARAFAC model fitting performance for EEMS subsets of increasing size and heterogeneity

ID	Species	Condition	Number of source samples	Dilutions	Factors	Core consistency	RSS
1	AME	LAB	3	12	2	99.4%	1.5 × 10^6^
2	AME	REF river	1	5	2	83.3%	2.9 × 10^6^
3	AME	POLLUT river	1	6	2	80.5%	2.6 × 10^6^
4	AME	LAB + REF river + POLLUT river	5	23	3	73.4%	8.1 × 10^6^
5	PIO	LAB	2	9	2	95.4%	2.5 × 10^6^
6	PIO + AME	LAB	5	20	2	99.2%	5.3 × 10^6^
7	PIO + AME	LAB + REF + POLLUT	7	32	3	88.3%	1.3 × 10^7^

AME: *A. metae*; PIO: *P. orinoquensis*; Lab: Laboratory; Ref river: Reference river; Pollut river: Polluted river; RSS = residual sum square

**Fig. 4:**
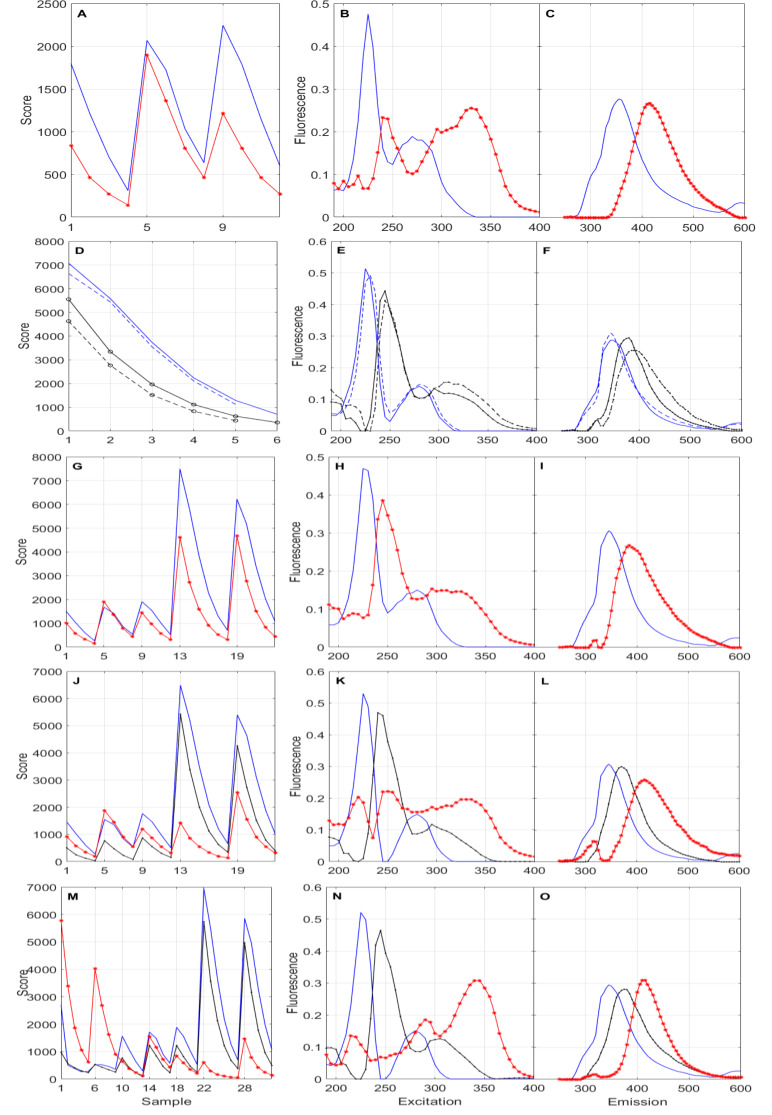
PARAFAC scores (proxy of fluorophore concentration) and factors (proxy of fluorophore EEMS) for various subsets and the whole EEMS set. The panels **A**, **B** and **C** show the scores, and excitation and emission spectral shapes, respectively, of the bile baseline fluorescence two-factor (1 & 3) model applied to the spectral surfaces of the three laboratory-conditioned *A. metae* pooled samples. Similarly, panels **D** through **F** show similar results of the bile baseline fluorescence two-factor (1 & 2) model but for the pooled samples of the reference and polluted rivers (dash line: REF; solid line: POLLUT). The panels **G** through **I** show scores and spectral shapes for the whole *A. metae* spectral dataset represented by two factors (1 & 3). Panels **J** through **L** show scores and spectral shapes for the whole *A. metae* spectral dataset represented by three factors (1, 2 & 3). Finally, panels M through O show scores and spectral shapes for the whole sample set including laboratory and field specimens of both species represented by three factors (1, 2 & 3). All the datasets consistently showed monotonic decrease of the scores with increasing dilution, as expected

**Fig. 5:**
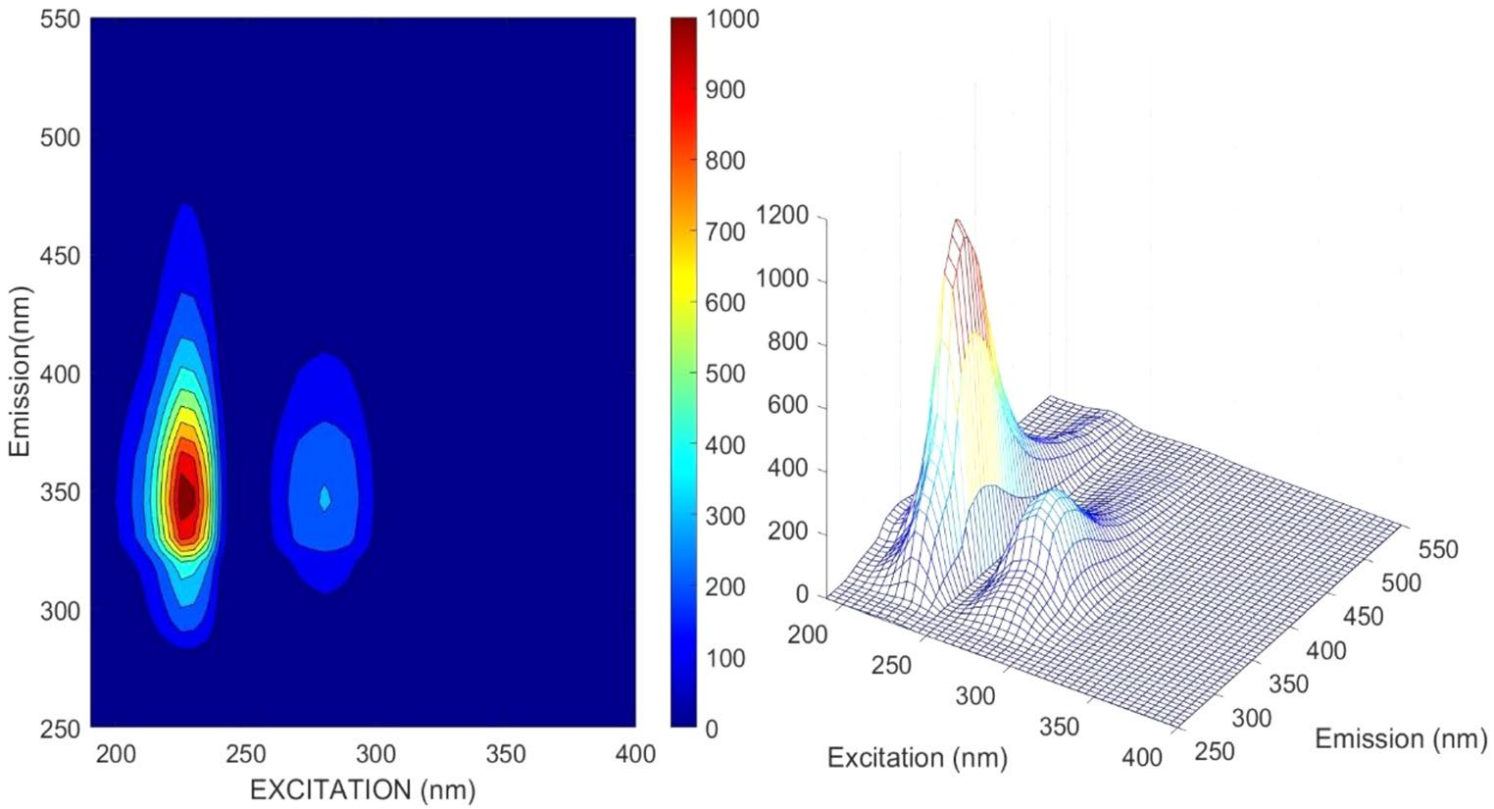
Fluorescence excitation-emission matrix of Factor 1 (tryptophan) as derived from PARAFAC

Figure 4 displays the scores and loadings for AME bile spectra, from laboratory-adapted specimens and those taken from rivers (both reference and polluted), along with those for the PIO laboratory-adapted specimen spectra. Overall, it is evident that scores decreased monotonically as samples were diluted, which indicates that concentration dilution (Eq. 1) dominated over any second order effects, particularly inner filter, and that the factor disaggregation accuracy improved as differently exposed AME individuals and a second species (PIO) were added to the analysis. The PARAFAC-retrieved Factor 1 (blue thick lines) showed remarkable persistence and stability among the different EEMS sets, despite scores being up to one order of magnitude larger for AME compared to PIO (Fig. 4M). Our database search spectrally identified this fluorophore as tryptophan [52, 53] (see Figure S9). Our PARAFAC-derived Factor 1 EEM surface is spectrally and wavelength-wise very similar to that of tryptophan as measured by Yang, et al. [54], which confirmed Factor 1 as tryptophan (Fig. 5).

Factor 1 (tryptophan) was more important than Factor 2 (black), and a lately added Factor 3 (red), for *A. metae*. The PARAFAC-retrieved spectral shape of Factor 2 (black) for AME LAB only samples (Fig. 4B-C) was significantly different than for the two river sample sets (REF and POLLUT – Fig. 4E-F). In contrast, Factor 2 was remarkably similar between the REF and POLLUT sample sets, which suggest that AME Factor 2 might be associated to natural media exposure. This was further strengthened by the fact that Factor 2 scores were ~ 3 times larger for AME in natural media than for laboratory-conditioned individuals as per a PARAFAC 2-factor analysis of the full (laboratory and field) *A. metae* dataset (Fig. 4G). A significant drop in core consistency, from > 80% to < 50% (Table 2), seemingly indicated that a third factor did not improve the PARAFAC representation of the full AME dataset, which might be reasonably well represented by two factors. AME Factor 3 spectral shape (red trace in Fig. 4K-L) anticipated that of a more robust, PIO-inclusive Factor 3 (Fig. 4N-O), which was ~ 6 times more abundant in the *P. orinoquensis* bile samples than in AME, according to scores (Fig. 4M). The addition of the PIO samples also improved the core consistency of the 3-factor PARAFAC from 73 to 88% (Table 2), which implies that the more abundant fluorophore (or group of fluorophores) in PIO (Factor 3) was also present in all the AME samples. Moreover, forcing the full dataset PARAFAC to two factors distorted the spectral shape of Factor 1 (see Fig S10), making it unrecognizable as tryptophan. Thus, three factors were needed to properly represent the full EEMS set. Our results vouch for PARAFAC’s ability to reduce complex spectra into its components provided the fed samples encompass enough variability, i.e. species and exposure, in our case. Future investigations should incorporate samples of PIO in natural media and of other fish species, along with chromatographic analyses for a more precise identification of the actual fluorophores.

**Fig. 6:**
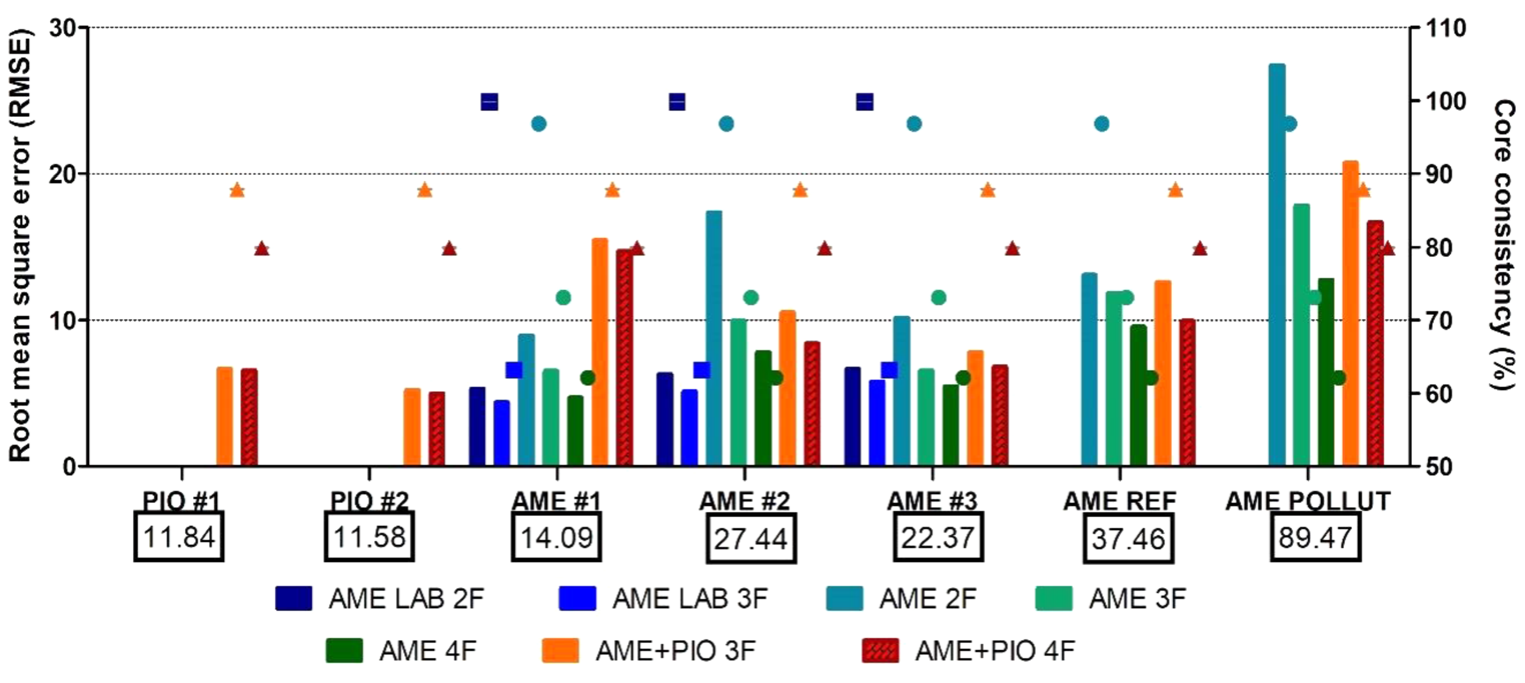
PARAFAC root mean square error (RMSE) and core consistency for each individual sample EEMS. RMSE is an absolute error metrics for the PARAFAC-estimated EEMS referred to the observed one, thus sensitive to the fluorescence intensity. In turn, core consistency is sensitive to EEMS subset variability and the number of factors. The RMSE is shown as bars scaled on the right axis. Core consistencies are shown as dots. AME LAB 2 F: *Aequidens metae* from laboratory with 2 factors; AME LAB 3 F: *Aequidens metae* from laboratory with 3 factors; AME 2 F: *Aequidens metae* from laboratory and rivers with 2 factors; AME 3 F: *Aequidens metae* from laboratory and rivers with 3 factors; AME 4 F: *Aequidens metae* from laboratory and rivers with 4 factors; AME + PIO 3 F: *Aequidens metae* from laboratory and rivers and *Piaractus orinoquensis* from laboratory with 3 factors; AME + PIO 4 F: *Aequidens metae* from laboratory and rivers and *Piaractus orinoquensis* from laboratory with 4 factors. Boxed data are the mean fluorescence signals

We also assessed the ability of PARAFAC to represent each individual sample EEMS. Figure 6 shows the PARAFAC representation accuracy, expressed as root mean square error (RMSE), and core consistency, for each EEMS as a function of the modeled EEMS set and number of factors (Table 2). The RMSE to mean fluorescence ratios were higher for the weakly fluorescent bile of lab-conditioned individuals (~ 0.3–0.6) than for the quite fluorescent bile of natural media exposed individuals (~ 0.25). This points to a hard to explain and remove residual in the baseline fluorescence. Overall, the most important RMSE and core consistency controlling factors were the EEMS set followed by the number of factors. In general, for a given dataset, increasing the number of factors reduced marginally the RMSE but also significantly the core consistency, which, according to Bro [44], must be close to 100% for the model to be valid. Thus, to preserve the core consistency, no more than two factors were required to represent the spectral surfaces of highly similar datasets (AME LAB, AME REF + POLLUT, PIO). As discussed above, only the full dataset required three factors. Also, remarkably, the RMSE of all the AME samples but AME #1 decreased upon modeling them together along with the PIO samples, while increasing the number of factors from 2 to 3. The “anomaly” implies that AME LAB #1 is less alike to PIO than the rest of AME, which confirms the individual-to-individual variability.

We were unable to find similar enough fluorescence spectra for a preliminary identification of Factors 2 and 3, although as argued above, Factor 2 is likely associated with food or other exposure factors present in natural media. *P. orinoquensis* is an omnivorous, voracious fish that is active throughout the water column. In contrast, *A metae* is a territorial but not voracious fish with benthic habits. Significant food and metabolic differences, thus bile composition [50], are thus expected between these two species, particularly for individuals in natural media, for which the concentrations of Factor 1 (tryptophan) and Factor 2 were significantly higher than for laboratory adapted individuals. A differentiation between species and individuals was also observed with time resolved fluorescence on EROD measurements [55].

The literature on fish bile composition and fluorescence is rather scarce [56]. Studies on human bile are obviously more abundant and could enlighten fish bile investigations. Recent studies evidenced that human gut microbiota regulates tryptophan metabolism and efficiently hydrolyzes bile salts, which yields several conjugated, potentially fluorescent compounds [57–59]. In addition, bile acids, such as taurocholic or cholic acid, play a vital role in hundreds of biological processes, resulting in a high degree of interaction with vitamins, fats, and other molecules [60]. Therefore, bile fluorescence can be affected by the oxidation state of these compounds, thus the bile composition at the sampling time.

### Score – Dilution Factor Relationships

**Fig. 7:**
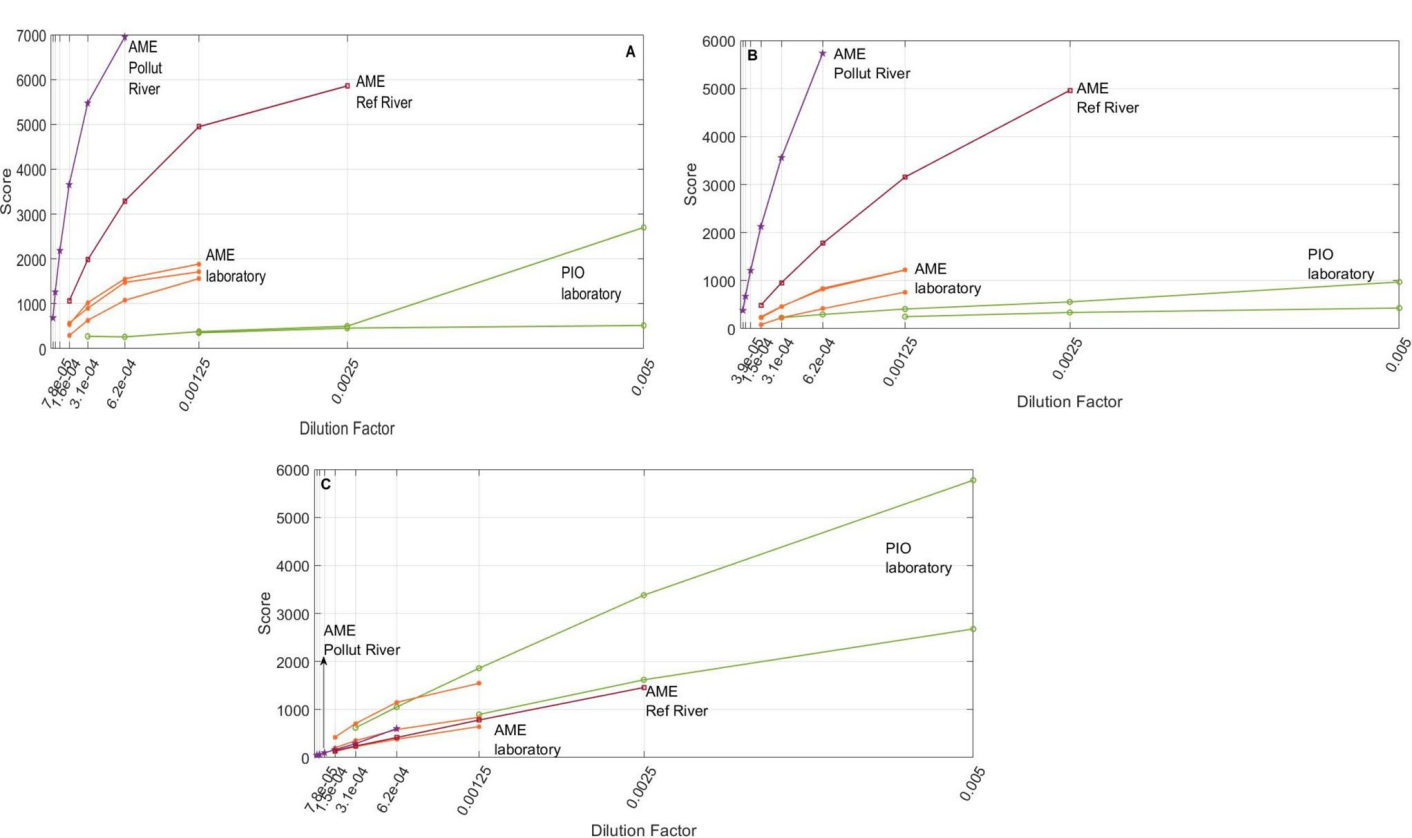
Score - dilution factor curves. (**A**) Factor 1 (shown in blue in Fig. 4), identified as tryptophan; (**B**) Factor 2 (shown in black in Fig. 4); (**C**) Third factor 3 (shown in red in Fig. 4)

Although the dilution factor (*DF*) is truly proportional to concentration, two samples with the same *DF* may have completely different concentrations of the same *k*-th fluorophore (*c*_*k*_) depending on their source sample concentration (*c*_*0,k*_). Plotting a factor score against *DF* allows for the identification of residual inner filter effects and the estimation of fluorophore concentration ratios among samples as fluorescence is a monotonic, univocal function of concentration in the absence of strong inner filter and other second-order effects. Residual inner filter non-linearities are clearly present in Factor 1, and to a lesser extent in, Factor 2 (Fig. 7), particularly in natural media individual samples. This suggests that samples are richer in Factor 1 (tryptophan) than in Factor 2, and that these two factors are stronger absorbers than Factor 3 or a combination of both. Comparing at an equal score (proxy of univocal fluorescence) of 1000, allowed us to estimate the source sample Factor 1 (tryptophan) concentration ratios as follows: AME POLLUT : AME REF : AME LAB : PIO ≈ 16 : 3 : 1 : 0.09. This means that Factor 1 concentration in bile was ~ 170 times higher in AME in a polluted river than in lab-conditioned PIO individuals. The estimated ratios for Factor 2 were quite similar: AME POLLUT : AME REF : AME LAB : PIO ≈ 21 : 4 : 1 : 0.25. This suggests that Factors 1 and 2 could be metabolically or chemically associated. Figure 7C revealed that all the source samples had comparable concentrations of Factor 3 (concentration ratios < 3). The misleading conclusion on higher concentrations of Factor 3 in PIO was artificially caused by the elimination from the analysis of the low dilution ratio and, high concentration AME samples due to strong inner filter effects from Factors 1 and 2.

## Conclusions

The bile fluorescence levels of the two tropical freshwater fish species investigated, *A. metae* (AME) and *P. orinoquensis* (PIO), were high, and their spectra were highly species-specific and highly variable intraspecies. A plain representation of the bile baseline fluorescence is thus unsuitable for these species.

The presence of inner filter effects down to 1:400 dilutions evidenced AME fluorophores as strong light absorbers, which suggests that a single specimen fluorescence analysis, i.e. without pooling, would be feasible for AME despite their low bile volume.

Major differences in fluorescence were found among various brands and grades of methanol and ethanol. The 1:1 v/v mixture of water with a spectrally selected analytical grade ethanol was successfully used as low volatility and low-cost bile solvent.

The PARAFAC analysis of incremental size and variability spectral subsets showed that our excitation-emission matrix spectra (EEMS) set can be properly decomposed into three factors, i.e. three fluorophores. This set included laboratory-adapted AME and PIO bile samples along with AME caught in apparently clean and polluted rivers. The three factors were present in all samples although in concentrations (scores) ranging over almost 3 orders of magnitude. Factor 1 was identified as tryptophan. Although the other two remain unidentified, tryptophan and Factor 2 are likely metabolically or chemically associated and are more abundant in natural media than in laboratory conditions. Factor 3 was ~ 6 times more abundant in PIO than in AME, and Factors 1 and 2 were significantly higher in natural media AME individuals than in their laboratory-adapted peers.

All the score-dilution ratio curves were monotonic and had a positive slope. Their analysis suggests that samples were richer in tryptophan than in Factor 2 and that these two factors are stronger light absorbers than Factor 3. It also allowed us to estimate that tryptophan and Factor 2 were ~ 5 times more concentrated in the bile of fish living in a polluted river than in a clean one, and ~ 2 orders of magnitude higher than in laboratory-adapted PIO individuals.

The bile baseline fluorescence spectra of AME and PIO were too intense and variable to be considered spectrally constant. We have demonstrated that the apparently unrelated EEMS of these species can be successfully reduced to a small number of common factors, one of which was identified as tryptophan. Thus, our results pave the way for the successful inclusion of bile matrix variable components into the spectral analysis of bile fluorescence for pollutant and other metabolite detection and quantification.

## Electronic Supplementary Material

Below is the link to the electronic supplementary material.

Supplementary Material 1

## Data Availability

Raw fluorescence data are available at https://doi.org/10.7910/DVN/TUKEWD. All other relevant data generated and analyzed during this study are available in the Supplementary Information.
