# Supplementary material for: Variability of Bile Baseline Excitation-emission Fluorescence of Two Tropical Freshwater Fish Species
Source: J Fluoresc. 2024 Sep 12;35(7):5413–27. doi: 10.1007/s10895-024-03871-x (PMC12325423; doi:10.1007/s10895-024-03871-x)
Supplement: Supplementary file 1 — Supplementary Material 1 [file 10895_2024_3871_MOESM1_ESM.pdf]

SUPPLEMENTARY MATERIAL

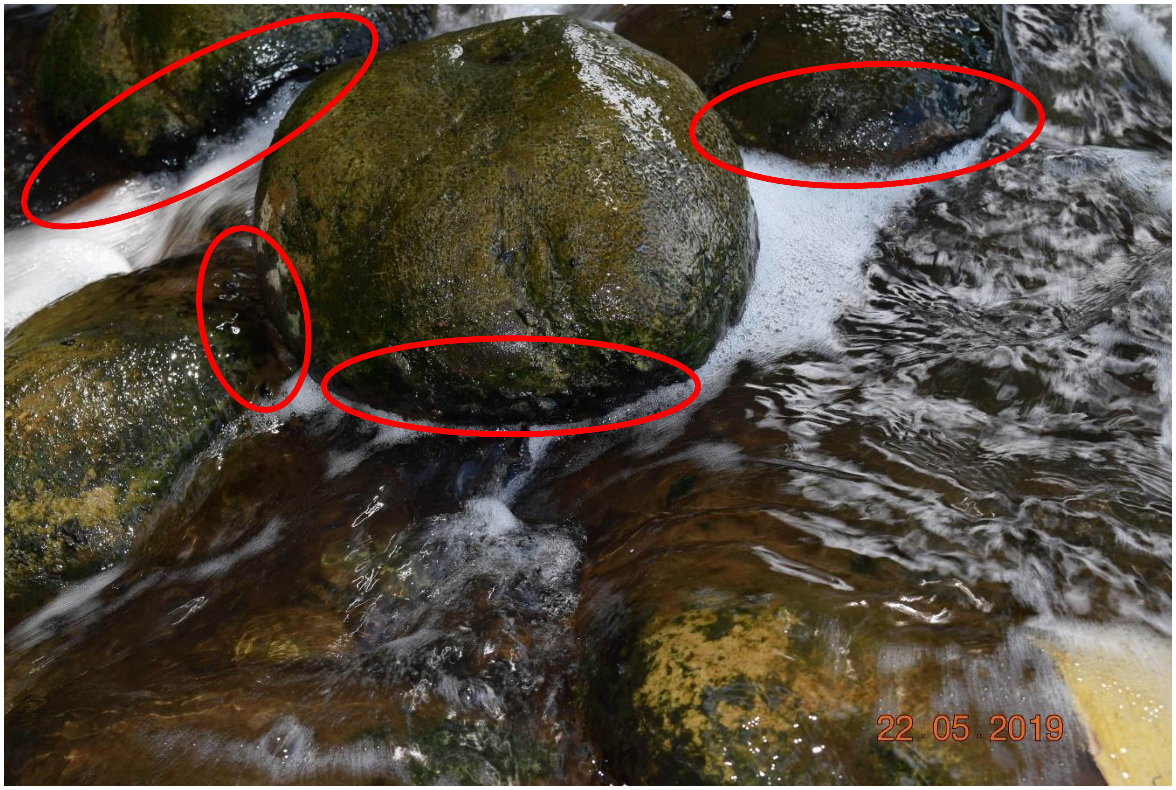

Figure S1. Crude oil microparticles adhered to riverbed stones near an oil production wastewater discharge point. Photo: Diego Mora-Solarte.

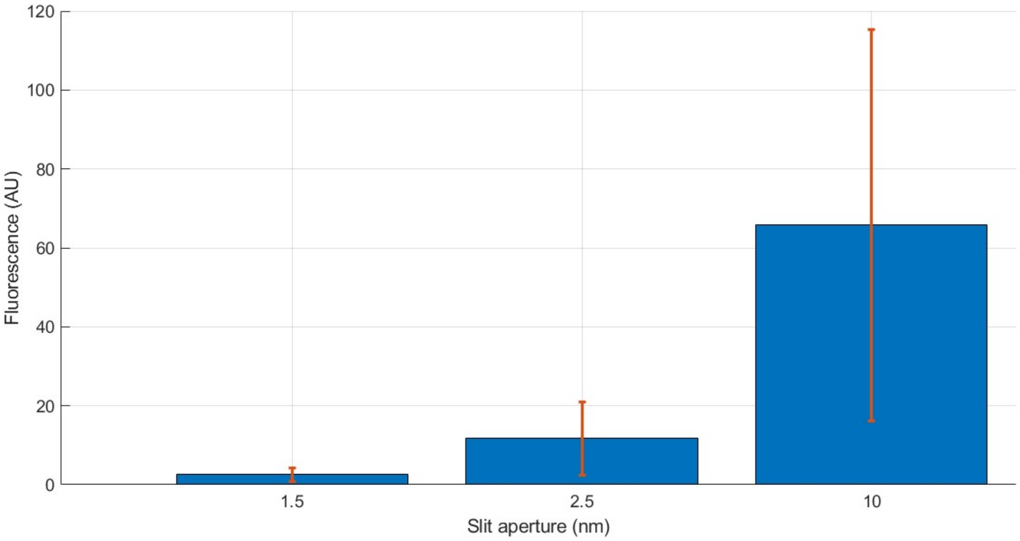

Figure S2. Excitation-emission matrix (EEM) average fluorescence of iso-propanol as a function of slit aperture at 1.5, 2.5, and 5 nm. Red bars are the standard deviations of fluorescence for each EEM.

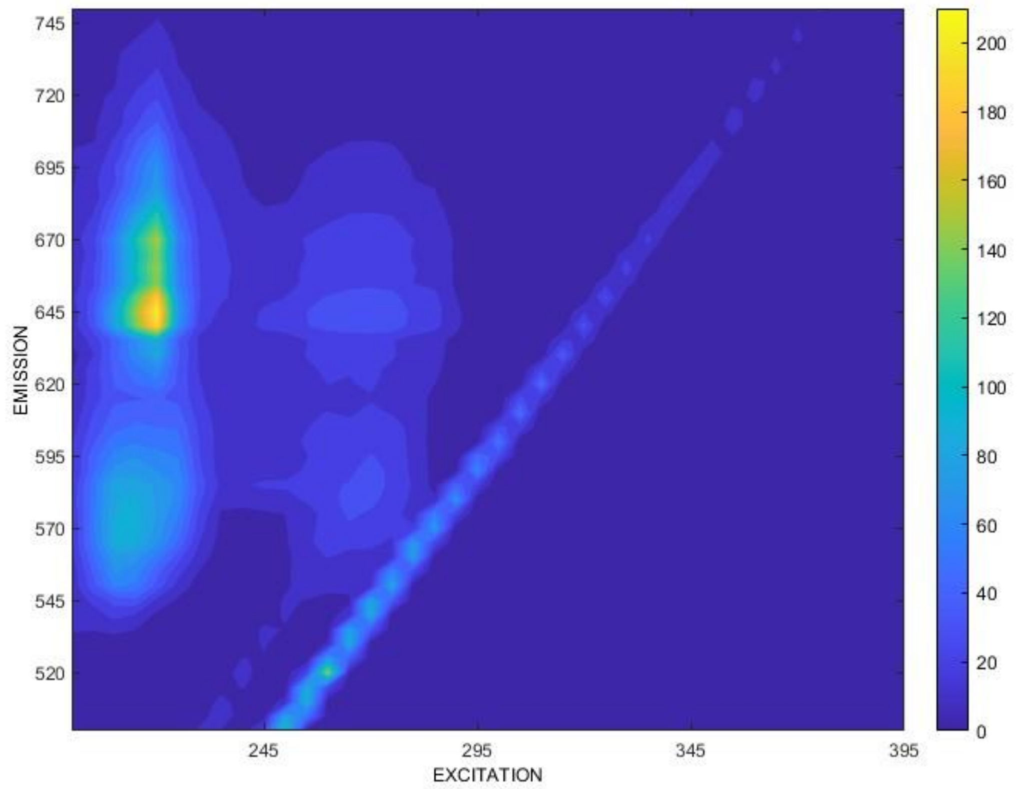

Figure S3. EEM of pure iso-propanol.

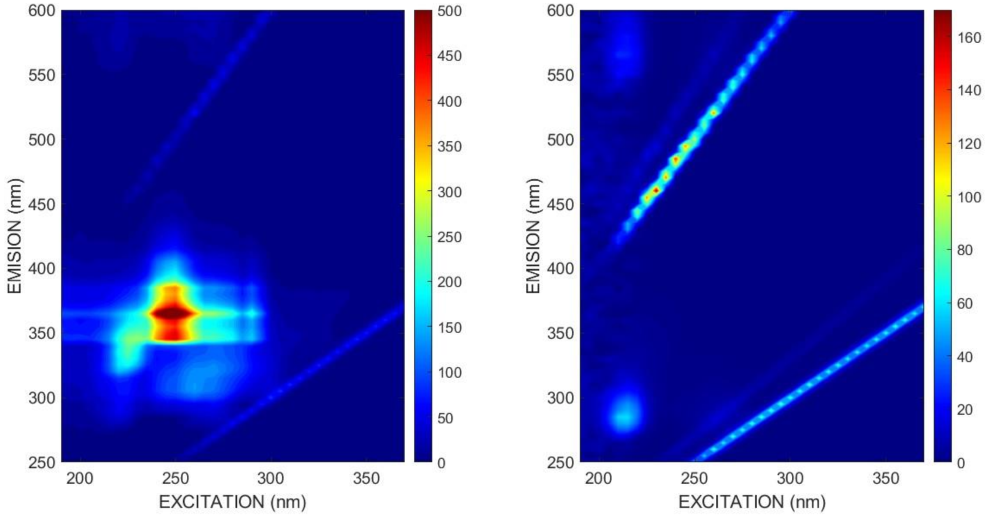

Figure S4. Excitation-emission matrix of methanol. Left: Analytical grade methanol (Merck). Right: HPLC grade methanol (JT Backer).

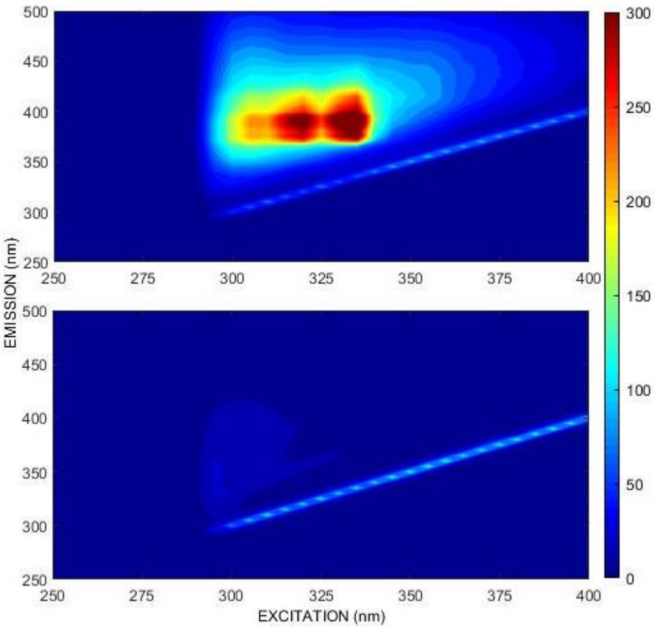

Figure S5. Top: Excitation-emission matrix of a 189-ppb pyrene in ethanol (Chemi) solution. Down: EEM of the ethanol brand used for the EEM above, i.e., EEM of pure Chemi ethanol.

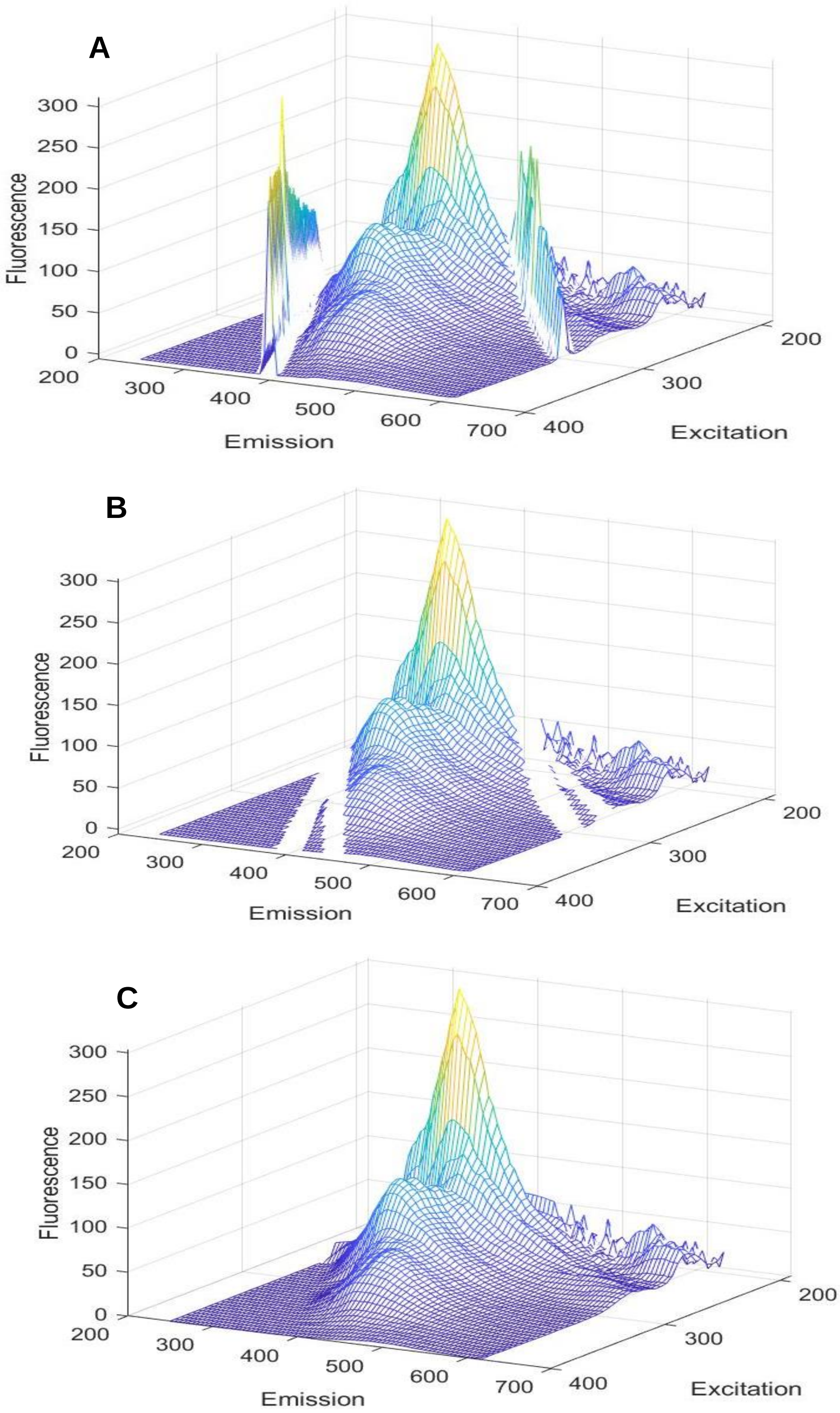

Figure S6. EEMS of a *A. metae* bile sample at *DF* = 0.00125. Panel A. Raw data. Panel B. After scattering removal. Panel C. Interpolated after scattering removal.

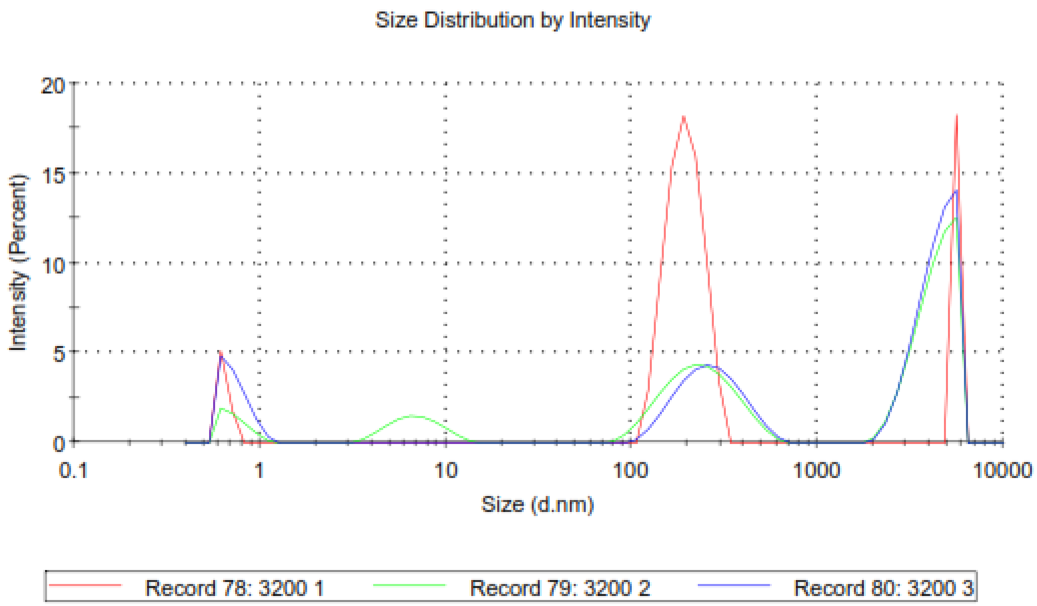

Figure S7. Particle size distribution in a bile sample at *DF* = 0.31x10^{-3}.

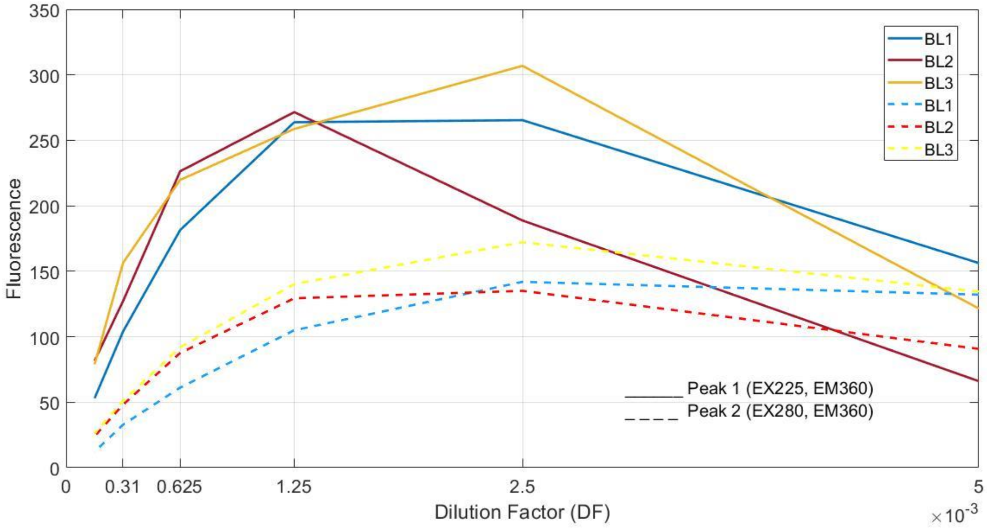

Figure S8. Dilution curves of three *A. metae* bile samples. Fluorescence was observed at their main excitation-emission peak (BL1-3, solid lines) and at their secondary excitation-emission peak (BL1-3, dashed lines). Strong inner filter effects were observed above *DF* = 1.25 x 10^{-3} and 2.5 x 10^{-3} for the main and secondary peaks,respectively.

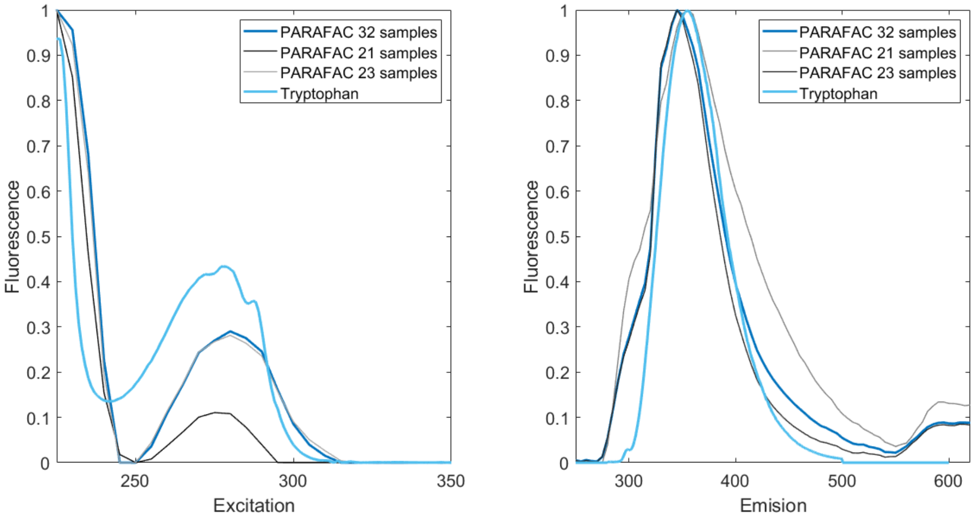

Figure S9. Spectral comparison of the various PARAFAC-derived versions of Factor 1 (blue factors in Figure 4) with the excitation (left) and emission (right) fluorescence spectra of tryptophan as reported on Search light™.

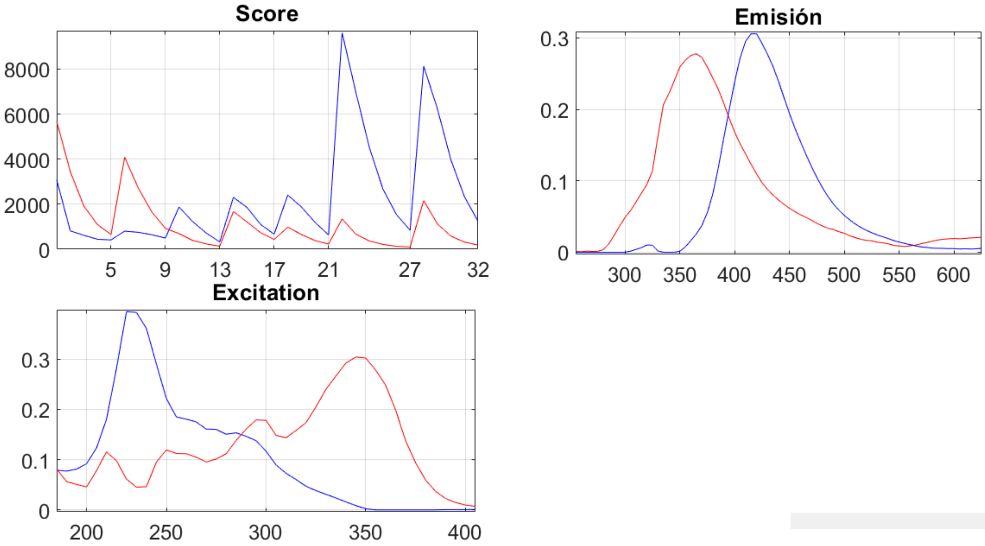

Figure S10. PARAFAC results for all the *A. metae* and *P. orinoquensis* samples using two factors only.
